# The Non-Covalent Interactions and In Vitro Radical Scavenging Activities of the Caseinate-Galangin and Caseinate-Genistein Complexes

**DOI:** 10.3390/antiox8090354

**Published:** 2019-09-01

**Authors:** Chun-Min Ma, Xin-Huai Zhao

**Affiliations:** Key Laboratory of Dairy Science, Ministry of Education, Northeast Agricultural University, Harbin 150030, China

**Keywords:** caseinate, galangin, genistein, non-covalent interaction, spectroscopy, molecular docking, antioxidation

## Abstract

Non-covalent interactions and in vitro radical scavenging activities of the complexes formed by the commercial milk protein product caseinate and one of the two polyphenols (galangin and genistein) were assessed by the multi-spectroscopic techniques, molecular docking, and detection of scavenging activities against the 1,1–diphenyl-2-picryl-hydrazyl (DPPH), 2,2′-azinobis(3-ethylbenzothiazoline-6-sulfonic acid) (ABTS), and hydroxyl radicals. The caseinate bound with the two polyphenols showed conformational changes and increased scavenging activities, compared with original caseinate. The caseinate-polyphenol binding was driven by the hydrophobic interaction and hydrogen-bonds, while hydrophobic interaction was the main binding force. Meanwhile, sodium dodecyl sulfate and urea could damage the essential hydrophobic interaction and hydrogen-bonds, respectively, and thus led to decreased apparent binding constants for the caseinate-polyphenol binding. Based on the measured values of several apparent thermodynamic parameters like ΔH, ΔS, ΔG, and donor–acceptor distance as well as the detected radical scavenging activity, galangin having more planar stereochemical structure and random B-ring rotation always had higher affinity for caseinate than genistein having location isomerism and twisted stereochemical structure, while the caseinate-galangin complex showed higher radical scavenging activity than the caseinate-genistein complex. It is thus concluded that both chemical and stereochemical structures of polyphenols are crucial to the affinity of polyphenols for protein and antioxidant activities of the protein-polyphenol complexes.

## 1. Introduction

Milk proteins are important sources of the nutrients and are major drivers in the structure formation of dairy products [1]. In bovine milk, caseins constitute about 80% of milk protein, and can be divided into four main types in terms of α_S1_-, α_S2_-, β-, and κ-caseins, with a mass ratio of about 4:1:4:1.3 [2]. The four casein fractions possess almost similar molecular weight (α_S1_-casein 23.62, α_S2_-casein 25.50, β-casein 24.09, and κ-casein 19.00 kDa) but different unfolding degrees [3,4]. These caseins adapt their structural changes in environmental conditions, and are present in native milk as supra-molecular aggregates so-called as casein micelles [1,5]. The casein micelles are very stable in native state, and have average diameter about 200 nm [6]. The agreed biological functions of casein micelles are to provide essential nutrients for the growth and energy requirements of the neonate and to prevent calcification of the mammary milk system [7]. Moreover, caseins are hydrophobic and rich in Pro residues, but only have a few Cys residues [8]. Thus, caseins have a tendency to binding with other food components, due to their hydrophobic character; for example, they can bind with several compounds such as vitamins [4], carotenes [2], and flavoniods [8]. Thus, a fundamental understanding of the generated complexes as a result of the interactions between caseins and other food components can not be ignored, from the scientific point of view.

Polyphenols are the food components that also are widely present in fruits, vegetables, tea, and legumes, and have many biological functions like anti-oxidation, anti-cancer, anti-cardiovascular disease [9,10]. Polyphenols are thus regarded as natural antioxidants, and also known having strong affinity for proteins [11]. Therefore, the interactions between polyphenols and proteins receive attention in the field of food science, because these interactions might bring about various changes in the properties of both proteins and polyphenols, such as their structures, bioactivity, and stability. For example, the structural properties of caseins were altered upon the interactions with tea polyphenols, reflected by percentage increases in random coil but percentage decreases in α–helix and β-sheet structures [12]. Sahu and coauthors found curcumin could interact with the low-polarity regions of casein micelles, while the casein–curcumin complex had higher cytotoxic effect on cervical cancer HeLa cells than the free curcumin [13]. However, it was proved that when epigallocatechin gallate was bound with casein micelles, the formed complexes had lower inhibition on the growth of colon cancer HT29 cells, compared with the free epigallocatechin gallate [11]. In another past study, it was also found that caseins could increase the thermal stability of quercetin and fisetin, due to the interactions of caseins with the two polyphenols [14]. Thus, an investigation of the interactions between polyphenols and caseins would help to control the properties of food components.

Galangin and genistein, two important polyphenols that are consumed by the human beings as part of their diets, have been reported to have various health benefits including their antioxidant, anti-diabetic, anti-inflammatory properties, and preventive effects on various cancers [15,16]. These health promotions of polyphenols are mainly due to their protection against the damaging action of free radicals [17]. However, the non-covalent interactions between galangin/genistein and caseins as well as the radical scavenging activities of the formed complexes have not been investigated yet. In this study, a commercial bovine protein product caseinate was selected, while multi-spectroscopic techniques (intrinsic fluorescence spectroscopy, ultra-violet spectroscopy, and three-dimensional fluorescence spectra) were used to explore the non-covalent interactions and forces involved in the formation of caseinate-polyphenol complexes. Noteworthy, sodium dodecyl sulfate (SDS) and urea were added to interfere with the non-covalent interactions and forces, aiming to further verify the forces involved. Moreover, the molecular docking technique was performed to estimate possible binding sites as well as interaction energy of galangin and genistein in two mainly casein fractions α_S1_-casein and β-casein. Finally, the radical scavenging activities of caseinate with or without the two polyphenols was measured and compared using the 1,1–diphenyl-2-picryl-hydrazyl (DPPH), 2,2′-azinobis(3-ethylbenzothiazoline-6-sulfonic acid) (ABTS), and hydroxyl radical scavenging methods. This study aimed to clarify the caseinate-galangin/genistein interactions and to reveal the important role of polyphenol structures in the caseinate-polyphenol interactions and antioxidation of the formed complexes.

## 2. Materials and Methods

### 2.1. Materials

Both galangin and genistein (purity ≥ 98%) were bought from Meilun Biotechnology Co., Ltd. (Dalian, Liaoning, China), while urea was obtained from Xilong Chemical Industry Co. Ltd. (Shantou, Guangdong, China). The caseinate (protein content 910.0 g/kg, dry basis), SDS, 1,1–diphenyl-2-picryl-hydrazyl (DPPH), and 2,2′-azinobis(3-ethylbenzothiazoline-6-sulfonic acid) (ABTS) were all brought from Sigma-Aldrich Co., Ltd. (St. Louis, MO, USA). Other chemicals were of analytical grade, while all solutions were prepared in distilled water and were fresh before using.

### 2.2. Preparation of Caseinate and Polyphenol Solutions

Both galangin and genistein were dissolved in anhydrous ethanol and then diluted by 50 mmol/L phosphate buffer solutions (PBS, pH 6.8) to yield polyphenol stock solutions of 1 mmol/L, which were preserved in the dark at 4 °C prior to uses. Caseinate was dispersed in this PBS to prepare caseinate stock solution of 15 µmol/L, based on a calculated average molecular weight of 23 kDa for caseins. Part of the 1 mmol/L polyphenol stock solutions was added into 15 µmol/L caseinate stock solution to yield various polyphenol concentrations (5–40 µmol/L) with final ethanol concentration less than 1%, because ethanol at the concentration less than 1% did not have any influence on the protein conformation and fluorescence quenching [18]. These mixed solutions were kept in a temperature-controlled water bath for 30 min at three temperatures (293, 303, and 313 K), and transferred into quartz glass cuvettes to measure their fluorescence intensity values immediately as below. To interfere with the caseinate-polyphenol interactions and involved forces, SDS and urea dissolved in this PBS were added to the mixed caseinate-polyphenol solutions at final concentrations of 5 mmol/L (SDS) and 4 mol/L (urea), respectively, and then also measured for fluorescence intensity values.

### 2.3. Fluorescence Spectroscopy Assay

Fluorescence spectra of all caseinate solutions in the absence or presence of galangin, genistein, SDS, and urea were done at the F-4500 fluorescence spectrophotometer (Hitachi, Kyoto, Japan) using 15 µmol/L protein concentration. The emission spectra were monitored in the wavelength range of 300–450 nm while the excitation wavelength was fixed at 280 nm. Slit and scan rate were set at 5 nm and 240 nm/min, respectively.

#### 2.3.1. Assay of Fluorescent Quenching Mechanism

There exist two major mechanisms in the process of fluorescence quenching, dynamic quenching (resulting from the collision between the fluorophore and quencher) and static quenching (resulting from the formation of a ground-state complex between the fluorophore and quencher) [19]. The Stern–Volmer Equation (Equation (1)) was utilized to determine the fluorescence quenching mechanisms of quenchers (galangin and genistein) to caseinate.

F_0_/F = 1 + Ksv·Q = 1 + Kq·δ0·Q, (1)

F and F_0_ represent the fluorescence intensities of caseinate with or without quencher addition, respectively. Q is quencher concentration, Ksv is the Stern–Volmer quenching rate constant which can be calculated by the plot of F_0_/F versus Q, while Kq is the quenching rate constant of the bimolecular. The δ0 is the bimolecular average life-time without quencher, with a value near 10^−8^ s [20]. When the values of Kq is greater than the maximum diffusion collision quenching constant 2 × 10^10^ L/(mol s), the quenching of fluorescence is mainly caused by a static quenching; otherwise, the quenching mechanism is dynamic quenching when quencher is bound to protein.

#### 2.3.2. Assays of Apparent Binding Constants and Binding Site Numbers

For static quenching, the apparent binding constant (Ka) and binding site numbers (n) for the protein-polyphenol interaction can be obtained from the following double logarithmic Equation (Equation (2)) [21].

lg[(F_0_ − F)/F] = lgK + n·lgQ, (2)

In this equation, F and F_0_ are the respective fluorescence intensity values with or without quencher, while Q is quencher concentration. The apparent binding constant (Ka) and binding site number (n) can be obtained from the intercept and slope data of the plot of lg[(F_0_ − F)/F] against lgQ, respectively.

#### 2.3.3. Assays of Apparent Thermodynamic Parameters

The apparent thermodynamic parameters are the main evidence to confirm the involved inter-molecular force. Apparent enthalpy change (ΔH), apparent entropy change (ΔS), and apparent free energy change (ΔG) were determined based on the Van’t Hoff Equation (Equation (3)) and the Gibbs–Helmholtz Equation (Equation (4)) [22], respectively.

lnKa = −ΔH/(R·T) +ΔS/R,(3)

ΔG = ΔH − T·ΔS,(4)

where, R is gas constant [8.314 J/(mol K)] while T is the experimental temperature. Ka is the apparent binding constant at a set temperature (293, 303, and 313 K). Both ΔH and ΔS can be obtained from the slope and intercept data of the plot of lnKa against 1/T according to Equation (3). There are four major non-covalent forces between small molecules and proteins: Hydrogen-bonds, hydrophobic interaction, the van der Waals force, and electrostatic interaction. If ΔH > 0 and ΔS > 0, hydrophobic force plays a major role; if ΔH < 0 and ΔS < 0, hydrogen-bonds and the van der Waals force are the main driving forces; if ΔH < 0 and ΔS > 0, electrostatic force is dominant [22].

#### 2.3.4. Assay of Efficiency of Energy Transfer

Energy transfer between small molecules and proteins can be determined using the Förster non-radiative energy transfer theory. For this theory, the energy transfer will occur between small molecules and proteins if the following two conditions are satisfied: (a) The donor itself (protein) can produce fluorescence and can overlap with the absorption of acceptor (polyphenol); (b) the distance between donor and acceptor is less than 7 nm. Energy transfer efficiency (E) from donor to acceptor is thus obtained using the Equation below (Equation (5)) [23].

E = 1 − (F/F_0_) = R_0_^6^/(R_0_^6^ + r^6^), (5)

F and F_0_ represent the fluorescence intensity values with or without polyphenol, and r is the distance of the donor and acceptor. R_0_ is the critical distance that 50% of the excitation energy is transferred to proteins and can be estimated according to the below Equation (Equation (6)).

R_0_^6^ = 8.8 × 10^−25^·K^2^·N^−4^·φ·J, (6)

where, K^2^ is a spatial factor of orientation and is related to the transition dipoles of acceptor and donor. N is the refractive index of medium, while φ is the fluorescence quantum yield of the donor. J is the overlap integral that expresses the effect of overlap between the emission spectrum of the donor and the absorption spectrum of the acceptor while concentrations of both donor and acceptor are equal (15 µmol/L), and can be calculated by the Equation below (Equation (7)).

J = ∑F(λ)·ε(λ)·λ^4^·dλ/∑F(λ)·dλ, (7)

where, F(λ) is the fluorescence intensity of the donor at wavelength λ, while ε(λ) is the molar absorption coefficient of the acceptor at wavelength λ. The curves of F(λ) and ε(λ) can be acquired using a non-linear curve fitting.

### 2.4. Assay of Ultra-Violet Spectroscopy

Ultra-violet spectra (250–400 nm) of galangin, genistein, caseinate, and caseinate-polyphenol complexes were recorded using a UV-2600 UV-Vis spectrophotometer (Shimadzu, Kyoto, Japan) equipped with 10 mm quartz cuvettes. The protein concentration was kept at 15 µmol/L, while polyphenol stock solutions were sufficiently mixed with the caseinate stock solutions to yield polyphenol concentrations of 5–40 µmol/L. The mixed solutions were kept at 293 K for 30 min, and then transferred into quartz glass cuvettes to scan their absorption spectra with a sampling interval of 0.1 nm immediately. The PBS was used to adjust baseline.

### 2.5. Assay of Three-Dimensional Fluorescence Spectra

Three-dimensional fluorescence spectra of the caseinate (15 µmol/L) and caseinate-polyphenol complex (15 µmol/L for both) were monitored at 293 K using the fluorescence spectrophotometer (Hitachi, Kyoto, Japan) and 10 mm quartz cuvette. The emission and excitation wavelengths were recorded from 210–400 and 210–350 nm, respectively. The slits of emission and excitation were both fixed at 5 nm while the scanning rate was set at 240 nm/min. Other parameters were the same as those used in the assay of fluorescence spectra.

### 2.6. Molecular Docking Investigation

The amino acid sequence of α_S1_-casein (GeneBank: AAA30429.1) and β-casein (GeneBank: AAA30431.1) were obtained from the NCBI database (Bethesda, MD, USA). The structures of α_S1_-casein and β-casein were predicted using the I-TASSER protein structure server (https://zhanglab.ccmb.med.umich.edu/I-TASSER). Out of the five predicted models by I-TASSER of the submitted sequence, the α_S1_-casein and β-casein models with the highest confidence score of −4.87 and −4.56 were chosen to carry out further docking [24]. The three dimensional structure files of galangin and genistein were also retrieved from the NCBI database of PubChem in SDF file format. The downloaded SDF format files were converted to PDB format files using the OpenBable GUI 2.4.1 software (Free Software Foundation Inc., Boston, MA, USA), which were then used in subsequent docking experiments.

Molecular docking was carried out using the AutoDock 4.2 package (Scripps Institution, San Diego, CA, USA). The PDB files of the ligands and receptors were modified by removing all water molecules, and then by adding hydrogen atoms and charges before docking. The grid box, with dimensions of 6 × 6 × 6 nm and 0.0375 nm space, was set using the AutoGrid program in this package. The Lamarckian Genetic Algorithm was used, while the numbers of genetic algorithm runs were set to 100 in the docking analysis [25]. Binding modes with the minimized interaction energy were selected as the actual binding mode. Docking image results were created using the Discovery Studio 3.0 Visualizer program (Accelrys Co., San Diego, CA, USA).

### 2.7. Assay of Antioxidant Capacity

The ABTS method reported by Re and coauthors [26] was performed with minor modification. Briefly, ABTS stock solution was prepared in water at 7 mmol/L. A stable ABTS radical solution was prepared via adding K_2_S_2_O_8_ solution (final concentration 2.45 mmol/L) to the ABTS stock solution at a ratio of 1:2 (*v/v*). The mixture was kept in the dark overnight for 12–16 h before use. The stable ABTS radical solution was diluted with ethanol to obtain a stable initial absorbance near 0.7 at 734 nm using an UV-visible spectrophotometer (UV-2401 PC, Shimadzu Co., Kyoto, Japan). Adding 0.5 mL of sample solutions to 5 mL of the diluted ABTS radical solution, the absorbance was measured after 6 min. The blank used the diluted ABTS radical solution and ethanol. The scavenging activity of ABTS radicals was calculated using the Equation expressed as scavenging activity (%) = 100 × (blank absorbance – sample absorbance)/ blank absorbance.

The DPPH scavenging radical activity was measured using a reported method [27] with slightly modification. Briefly, one milliliter of 200 μmol/L DPPH solution (dissolved in ethanol) and 2 mL sample solutions were mixed and reacted for 30 min in the dark at 20 °C. The absorbance was measured using the same spectrophotometer at 517 nm with a blank containing DPPH solution and ethanol. The scavenging activity of DPPH radicals was calculated using equation expressed as scavenging activity (%) = 100 × (blank absorbance – sample absorbance)/ blank absorbance.

The scavenging capacity of hydroxyl radicals was determined as previously described [28]. The same volumes of the sample solution, 6 mmol/L FeSO_4_, and 6 mmol/L H_2_O_2_ were mixed and reacted for 10 min at 20 °C. After reaction, 6 mmol/L salicylic acid solution (dissolved in ethanol) of 1 mL was added and incubated at 37 °C for 30 min. The absorbance of the mixture was recorded at 510 nm using the same spectrophotometer, while the scavenging activity of hydroxyl radicals was estimated using equation expressed as scavenging activity (%) = [1 − (A_sample_ − A_control_)/A_blank_] × 100%. Where, A_sample_ is the absorbance of tested samples (the mixture with samples), A_blank_ is the absorbance of blank samples (the mixture without the samples), while A_control_ is the absorbance of control sample (the mixture without H_2_O_2_).

### 2.8. Statistical Analysis

Statistical analyses were carried out using the SPSS 17.0 software (SPSS Inc., Chicago, IL, USA). Origin 8.0 (OriginLab Co., Northampton, MA, USA) was used to perform non-linear curve fitting. For all experiments, three replicates were taken and the reported data were shown as means or means ± standard deviations. The differences between the means were analyzed by one-way analysis of variance (ANOVA) with Duncan’s multiple range tests at a significance level of 0.05.

## 3. Results

### 3.1. The Non-Covalent Interactions between Caseinate and Galangin/Genistein

**Figure 1:**
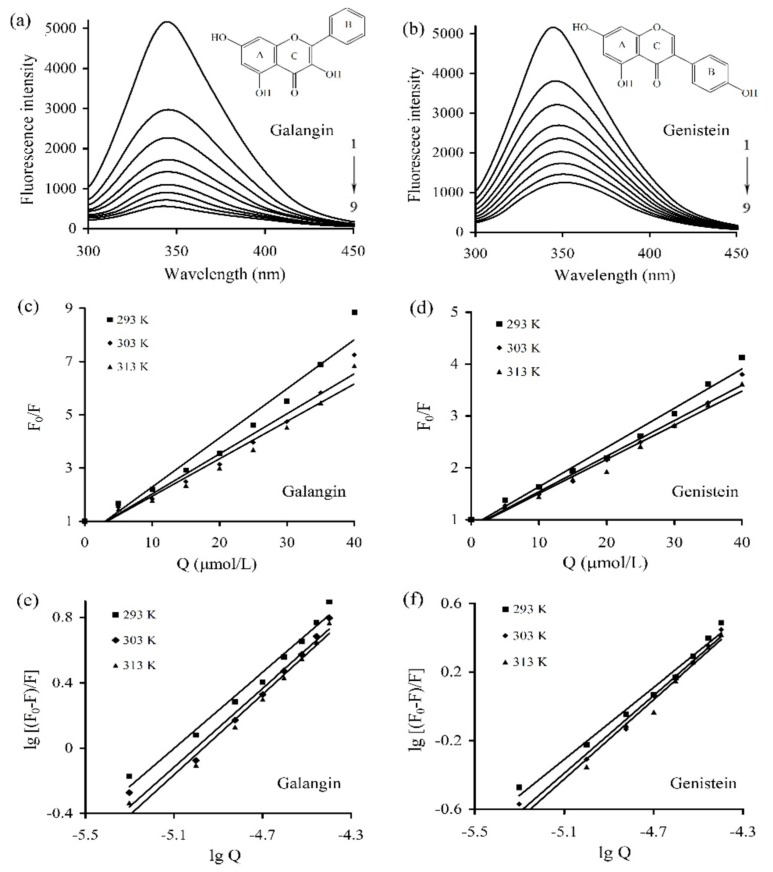
Fluorescence spectrum (300–450 nm) of caseinate solution with or without galangin (**a**) and genistein (**b**) at 293 K, the Stern–Volmer plots for galangin (**c**) or genistein (**d**), as well as the lg[(F_0_ − F)/F] versus lgQ plots for the binding of galangin (**e**) or genistein (**f**) with caseinate at the three temperatures. Caseinate was used at 15 μmol/L, while galangin/genistein was used at 0, 5, 10, 15, 20, 25, 30, 35, and 40 μmol/L (from 1 to 9).

The fluorescence emission spectra of caseinate solution containing galangin/genistein of different concentrations were measured under 293, 303, and 313 K. The obtained spectra under 293 K are depicted in Figure 1a,b (the curves under 303 K and 313 K are not shown). The measured fluorescence intensity of caseinate gradually decreased, accompanying concentration increases of galangin/genistein. The result thus showed that both galangin and genistein were bound to caseinate via the non-covalent interactions, which brought about fluorescence quenching of caseinate in a concentration-dependent manner.

The Stern–Volmer relation (Equation (1)) was used to elucidate the detailed quenching mechanism involved in the caseinate-polyphenol interactions. A plot of F_0_/F versus Q and the parameters of Kq and Ksv at three temperatures are given in Figure 1c,d and Table 1, respectively. The plot of F_0_/F versus Q was linear (coefficients > 0.95) (Figure 1c,d), indicating the quenching mechanism between galangin/genistein and caseinate, in theory, was static or dynamic. The values of Kq ranged from 6.61 × 10^12^ to 18.6 × 10^12^ L/(mol s), far larger than 2 × 10^10^ L/(mol s). It is thus concluded that the quenching of galangin/genistein to intrinsic fluorescence of caseinate was static quenching. It also could be seen from Table 1 that the corresponding Ksv value decreased when the temperature increased, indicating again that the quenching mechanism was static one.

The values of Ka and n were obtained from the double logarithmic regression curves (Figure 1e,f), and are listed in Table 2. The Ka values for galangin ranged from 8.83 × 10^5^ to 1.18 × 10^6^ L/mol, larger than those for genistein (1.02 × 10^5^–2.44 × 10^5^ L/mol). Thus, based on the measured binding constant, the affinity of galangin for caseinate was much stronger than that of genistein. Besides, the measured n values (close to 1) suggested that caseinate only had a single binding site to accommodate galangin or genistein molecules. The binding constants of the caseinate-polyphenol complexes in the presence of SDS or urea were also measured at 293 K and are given in Table 3. When SDS and urea was added, the Ka values for the caseinate-galangin interaction decreased to 7.40 × 10^5^ and 8.32 × 10^5^ L/mol, while those for the caseinate-genistein interaction also reduced to 8.60 × 10^4^ and 8.83 × 10^4^ L/mol, respectively. The decreased binding constants proposed that both SDS and urea were capable of damaging the caseinate-polyphenol binding via interfering with the essential non-covalent interactions.

The apparent thermodynamic parameters (ΔH, ΔS, and ΔG) for the caseinate-polyphenol interactions were listed in Table 2. The values of ΔH and ΔS were 11.0 kJ/mol and 151.5 J/(mol K) for the caseinate-galangin interaction, while those values were 33.4 kJ/mol and 209.7 J/(mol K) for the caseinate-genistein interaction. The positive values of ΔH and ΔS indicated the hydrophobic force was the major force for the assessed caseinate-polyphenol complexes. At these three temperatures, the ΔG values were −33.3, −34.9, and −36.4 kJ/mol for the caseinate-galangin interaction, while those values were −28.1, −30.2, and −32.3 kJ/mol for the caseinate-genistein interaction. The negative values of ΔG indicated that the binding process of caseinate and the two polyphenols was a spontaneous one. Moreover, based on the different ΔG values, caseinate would form more stable complex with galangin than genistein.

**Figure 2:**
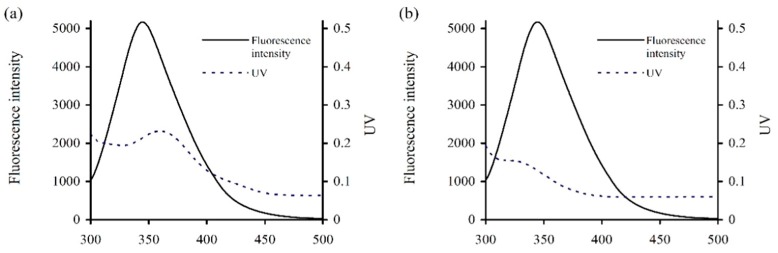
Overlap features of the fluorescence emission spectra of caseinate and ultra-violet (UV) absorption spectra of galangin (**a**) or genistein (**b**). Caseinate and galangin/genistein were used at 15 μmol/L.

From the fluorescence quenching results above (Table 2), it was suggested that energy transfer phenomenon might occur between caseinate and the two polyphenols. According to the Förster’s non-radiative energy transfer theory, energy transfer will occur efficiently when suitable spectral overlap is created between the targeted small molecule and macro-molecule. The spectral overlap between the emission spectrum of caseinate and the absorption spectrum of galangin or genistein were thus measured and are given in Figure 2. In general, *K*^2^ = 2/3, *φ* = 0.2, and *n* = 1.336. The apparent parameters E, R_0_, r, and J were thereby calculated (Table 4). The distances between caseinate and the two polyphenols (i.e., r values) were 1.56 and 1.42 nm (<7 nm), confirming the energy transfer from caseinate to galangin/genistein and the interactions between caseinate and galangin/genistein.

### 3.2. Secondary Conformation Changes of Caseinate induced by the Non-Covalent Interactions

**Figure 3:**
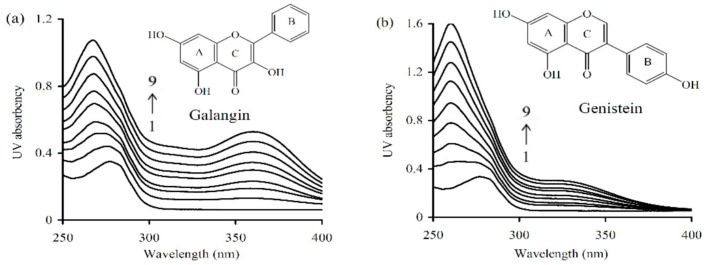
Absorption spectrum of caseinate solution without or with galangin (**a**) or genistein (**b**). Caseinate was used at 15 μmol/L, while galangin/genistein was used at 0, 5, 10, 15, 20, 25, 30, 35, and 40 μmol/L (from 1 to 9).

Ultra-violet (UV) spectra (Figure 3) provided an insight into the changes in secondary conformation of caseinate upon its interaction with the two polyphenols of different levels. Caseinate solution itself had an absorption peak around 277.4 nm. When the two polyphenols were added into caseinate solution, the wavelength of the absorption peak showed blue-shifting mode (toward the shorter wavelength region). Using 40 μmol/L galangin and genistein, the detected absorption peak shifted into 267.4 and 260.1 nm, respectively. In total, the intensity of absorption peak showed increasing trend while the peak position was shifted into shorter wavelength, accompanying the increasing levels of galangin and genistein. These results implied that caseinate could interact with galangin and genistein, and the resultant non-covalent caseinate-polyphenol binding induced conformational changes in caseinate.

The 3D fluorescence spectra were also used to explore the conformational change of caseinate. As shown in Table 5, the spectra of the caseinate and caseinate-galangin/genistein complexes showed two peaks. The peak 1 (λex/λem, 240/345 nm) revealed the spectral properties of the polypeptide backbone while the peak 2 (λex/λem, 285/345 nm) was the spectral characteristics of tryptophan (Trp) and tyrosine (Tyr) residues in caseinate. Both galangin and genistein induced the changes of peak positions and decreased values of peak intensity. The two peaks also showed a red-shifting about 5 nm because λem values were enhanced from 345 to 350 nm. These results indicated that the Trp/Tyr residues in caseinate were exposed to the environment. That is, caseinate had changed secondary conformation. It is also seen from these data that the quenching effect of galangin was larger than that of genistein (fluorescence intensity 241.9 versus 255.1 in peak I, or 1508 versus 2342 in peak II), showing result consistence with those obtained from the fluorescence experiments (Figure 1).

### 3.3. The Binging Sites and Interaction Energy of Caseinate-Pophenol Interactions

**Figure 4:**
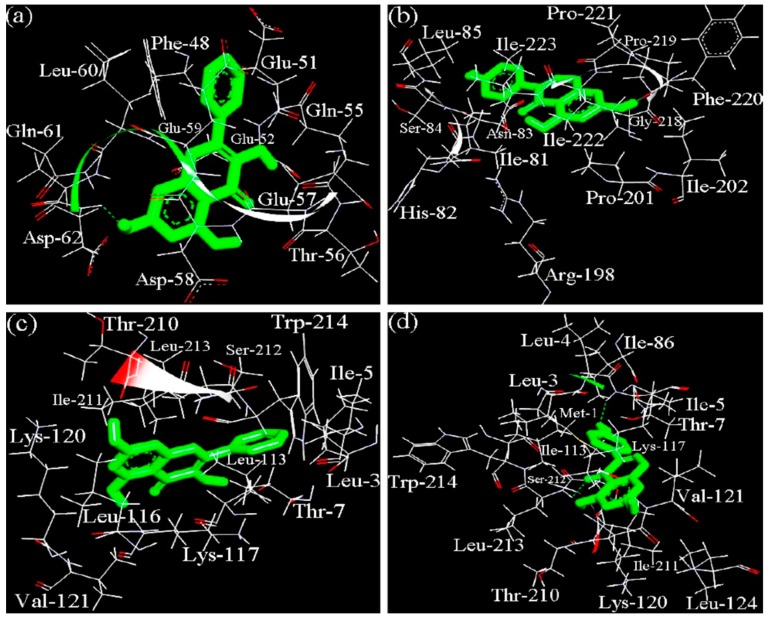
The docked sites for the non-covalent binding of β-casein (**a**,**b**) or α_S1_-casein (**c**,**d**) with galangin (**a**,**c**) or genistein (**b**,**d**). Hydrogen-bonds are shown by green dashes.

Molecular docking is an effective method that uses interaction energy assessment to predict the binding affinity and preferred orientation of small molecules to the targeted macromolecules, when they are bound to each other to form a stable complex. The two major fractions of bovine caseins, α_S1_-casein and β-casein, were thereby docked with galangin and genistein, to further estimate the preferred binding sites and interaction energy. In the actual binding modes with the lowest binding energy, the center coordinates (x, y, z) of these search space, for targeted proteins were 55.564, 60.728, and 82.310 for β-casein-galangin, or 60.470, 51.023, and 60.814 for β-casein-genistein, or 75.998, 65.667, and 54.382 for α_S1_-casein-galangin, or 75.998, 65.667, and 64.330 for α_S1_-casein-genistein, respectively. The 3D dominating conformations of the caseinate-polyphenol complexes, which had the lowest binding free energy, are shown in Figure 4, while the docking parameters are listed in Table 6.

The results indicated that binding of galangin and genistein to their binding sites in α_S1_-casein and β-casein was mediated through both hydrophobic and hydrogen-bond interactions, while hydrophobic interaction was the dominated force. It was also shown that α_S1_-casein had different binding sites than β-casein when interacting with the two polyphenols. In the β-casein-galangin complex, galangin might be surrounded by 11 amino acid residues including Phe-48, Glu-51, Glu-52, Gln-55, Thr-56, Glu-57, Asp-58, Glu-59, Leu-60, Gln-61, and Asp-62. This site provided one hydrogen-bond interaction, where a –OH group of galangin interacted with the nitrogen from Asp-62 of β-casein. In the β-casein-genistein complex, genistein might be surrounded by 14 amino acid residues including Ile-81, His-82, Asn-83, Ser-84, Leu-85, Arg-198, Pro-201, Ile-202, Gly-218, Pro-219, Phe-220, Pro-221, Ile-222, and Ile-223. The main force was also hydrophobic interaction; however, the –OH group of genistein had hydrogen-bond interaction with the oxygen from Pro-219 of β-casein. Therefore, β-casein provided different binding sites to galangin and genistein. In the α_S1_-casein-galangin complex, galangin might be surrounded by 13 amino acid residues like Leu-3, Ile-5, Thr-7, Leu-113, Leu-116, Lys-117, Lys-120, Val-121, Thr-210, Ile-211, Ser-212, Leu-213, and Trp-214. In the α_S1_-casein-genistein complex, genistein was surrounded by 16 amino acid residues including Met-1, Leu-3, Leu-4, Ile-5, Thr-7, Ile-86, Leu-113, Lys-117, Lys-120, Val-121, Leu-124, Thr-210, Ile-211, Ser-212, Leu-213, and Trp-214. Comparison results showed that both galangin and genistein could be bound into α_S1_-casein via very similar binding site or pocket, but two –OH groups of galangin might interact with the nitrogen from Ile-5 and Leu-213 of α_S1_-casein. Moreover, the docking results suggested that galangin had higher interaction energy (i.e., lower ΔG value) than genistein when the two polyphenols were docked with the same milk protein molecule.

### 3.4. Impact of the Non-Covalent Interactions on Radical Scavenging Activity of Caseinate

The measured radical scavenging activities of caseinate and the caseinate-polyphenol complexes with three polyphenol levels are reported in Table 7. The data showed that these caseinate-polyphenol complexes consistently had higher radical scavenging activities than caseinate itself. In addition, galangin with higher binding affinity for caseinate brought about higher scavenging activities for these caseinate-polyphenol complexes. However, genistein only resulted in slight increases in these scavenging activities for these caseinate-genistein complexes. Moreover, these caseinate-polyphenol complexes totally showed higher abilities to scavenge the hydroxyl radicals other than the DPPH and ABTS radicals. Overall, the non-covalent interactions between caseinate and the two polyphenols led to increased in vitro antioxidation for caseinate.

## 4. Discussion

When investigating the non-covalent binding of small molecules and proteins, fluorescence spectroscopy is a useful tool to provide a few of binding information. Fluorescence quenching refers to the decrease of the quantum fluorescence yield of a fluorophore induced by a variety of molecular interactions with the quencher molecules [12]. Intrinsic fluorescence of proteins is contributed by these hydrophobic amino acid residues (mainly Trp). Trp accounts for 1.7% of amino acid composition of caseins, while one molecule of α_S1_-, α_S2_-, β-, and κ-casein contains 2, 2, 1, and 1 Trp residues, respectively. Thus, the fluorescence of caseinate resulted from a combination of these casein fractions [26]. When caseins are bound with other small molecules, changed Trp fluorescence may be observed, which is depended on the impact of resultant interaction on protein conformation [12]. In this study, the fluorescence of caseinate was quenched by the two polyphenols galangin and genistein. These observed result were consistent with other reported results, in which the interactions between milk protein (α-casein and β-casein) and various polyphenols (such as tea polyphenols, resveratrol, genistein, and curcumin) were investigated [12,22,29]. The detected fluorescence quenching in this study suggested that galangin/genistein molecules were transferred from the polar phase (i.e., aqueous solution) to non-polar hydrophobic pockets of the caseins [22]. Moreover, it was measured that the distance from caseinate to the bound polyphenols was much shorter than 7 nm. On the basis of the Förster non-radiative energy transfer theory, this short distance ensured the significant energy transfer and interaction between caseinate and the two polyphenols.

The hydrogen-bonds and hydrophobic interaction are the two major forces in the non-covalent protein–phenol interactions. For instance, the interaction between tea flavonoids and milk proteins belonged to the hydrophobic interaction [8]. When apigenin and naringenin were bound onto β-lactoglobulin, the predominant force was van der Waals force and hydrogen-bonds; however, when genistein and kaempferol were bound onto β-lactoglobulin, hydrophobic interaction was the main force [30]. Both hydrogen-bonds and hydrophobic interaction were involved in the binding of resveratrol and sodium caseinate [19]. In this study, the disclosed binding forces were mainly hydrophobic interaction and in less extent hydrogen-bonds, while the binding process of caseinate and galangin/genistein was spontaneous (with negative ΔG values). From a chemical point of view, hydrophobic hydration of caseinate was destroyed when galangin or genistein interacted with caseinate; subsequently, part of the bound water was released into the medium and existed as the free water; thus, the whole process had positive ΔS value [31]. Moreover, urea as a hydrogen-bonds receptor is able to damage the hydrogen-bonds, while SDS as an anionic detergent is powerful to destroy the hydrophobic interaction [14,32]. Hence, urea and SDS were potential to damage the respective hydrogen-bonds and hydrophobic interaction in the complexes produced by caseinate and galangin/genistein. It was reasonable that adding the two chemicals in the systems led to decreased apparent binding constants for the caseinate-polyphenol complexes. Here, for the first time, SDS and urea were used as two interesting chemicals to verify the involved non-covalent interactions between small molecules and proteins.

Polyphenols can induce conformational changes for proteins via the protein-polyphenol binding. For instance, the secondary conformation of caseinate was altered upon its bindings with four tea polyphenols (catechin, epicatechin, epigallocatechin, and epigallocatechin gallate), reflected by a reduction in the α-helix, β-sheet structures but an increase in the random coil structure [12]. Polyphenols were also reported to change the secondary and tertiary structures of β-lactoglobulin [33]. The theaflavin from black tea at higher concentration could unfold the secondary structure of bovine serum albumin [34]. At pH 1.2, the non-covalent interactions between egg white proteins and tea polyphenols led to increased random and β-sheet structures [35]; however, the non-covalent interactions at pH 7.5 caused ordered secondary structure in egg white proteins [35]. In general, when polyphenols are bound to the hydrophobic pockets of proteins, protein structure will be changed, leading to possible protein unfolding and functionality changes [8]. Thus, the non-covalent interactions assessed in this study also induced conformational changes for caseins.

Different features of galangin and genistein might result in galangin with higher affinity for caseinate than genistein. Galangin and genistein have the same molecular weight and –OH number; however, they are different in chemical especially stereochemical features. Compared with galangin (B-ring in C-2), genistein (B-ring in C-3) shows a location isomerism of B-ring, which might confer genistein weaker binding affinity for caseinate. This result was consistent with a previous study [30], in which β-lactoglobulin showed higher affinity for apigenin (B-ring in C-2) than genistein. In addition, another previous study also indicated that the existence of −OH or other groups in the B-ring of flavonoids will twist or rotate the B-ring due to the steric hindrance, leading to twisted planar stereochemical structure and weakened interact with DNA [36]. It is thus recognized that these flavonoid molecules with near-planar structures are more easily to enter the hydrophobic pockets of proteins [37,38]. Genistein has one –OH group in its B-ring, while galangin does not have any –OH group in its B-ring. In theory, galangin has less steric hindrance, possesses more ideal planar stereochemical structure than genistein, and therefore could rotate randomly to an ideal angle in the hydrophobic pockets of caseinate (which was beneficial for the formation of caseinate-galangin complex) (Figure 4). Galangin thus was measured with higher affinity for caseinate than genistein.

Various radicals are considered as one of the major factors of several diseases and other health disorders. The non-covalent interactions between proteins and polyphenols have been extensively assessed in relation to the change of radical scavenging activity. Several past studies focused on the potential impacts of these interactions on radical scavenging activity of polyphenols themselves instead of proteins. It was found that the interaction between epigallocatechin gallate and β-casein caused decreased scavenging activity against the ABTS radicals, compared with the free epigallocatechin gallate [39]. The scavenging activity against the ABTS radicals of different caffee brews was also decreased by the addition of milk [40]. However, Almajano and coauthors had reported that the scavenging activity of α-casein and β-casein against the ABTS radicals at 30 °C increased with storage time because of the protein-epigallocatechin gallate interactions [41]. Similarly, this study found the caseinate-polyphenol complexes had better radical scavenging activities than the original caseinate. That is, these bound polyphenols endowed caseinate an enhanced antioxidation. Also, different antioxidative properties of galangin and genistein made a contribution to the activity differences for these caseinate-polyphenol complexes. It has been suggested that torsion angle of the B-ring of flavonoids with respect to the rest of the molecule strongly has an influence on the radical scavenging activities of flavonoids [42]. In detailed, the flavonols with planar structure possess higher radical scavenging activity, whilst those with slightly twisted structure will have weaker radical scavenging activity [42]. Thus, galangin with near-planar structure brought about much increased radical scavenging activity to the corresponding caseinate-galangin complex than genistein with twisted structure.

## 5. Conclusions

Taken together, the results of this study showed that caseinate could form corresponding complexes with two polyphenols (galangin and genistein) driven by the non-covalent interactions (hydrophobic interaction and hydrogen-bonds), while hydrophobic interaction was the mainly binding force. Both urea and SDS reduced the caseinate-polyphenol interactions by damaging hydrophobic interaction and destroying hydrogen-bonds, respectively. The caseinate-polyphenol interactions also led to changed secondary conformation in caseinate, and brought about increased radical scavenging activities for the caseinate-polyphenol complexes. Compared with genistein, galangin had higher affinity for caseinate and endowed the caseinate-galangin complex with increased radical scavenging activities. The location isomerism and stereochemical structure of the two polyphenols were proposed to have influence on polyphenols’ affinity for caseinate and increased radical scavenging activity of the caseinate-polyphenol complexes, highlighting an interesting role of chemical and stereochemical structures of polyphenols in the non-covalent protein-polyphenol interaction and radical scavenging activity of the formed complexes.

## Figures and Tables

**Table 1:** Stern–Volmer quenching constants (Ksv), quenching rate constants (Kq), and linear equations for the non-covalent caseinate-polyphenol interaction at three temperatures.

Polyphenols	T (K)	Equation	Ksv (10^4^ L/mol)	Kq [10^12^ L/(mol s)]	*R* ^2^
Galangin	293	Y = 0.1862Q + 0.410	18.6 ± 0.10 ^a^	18.6 ± 0.10 ^a^	0.953
	303	Y = 0.1511Q + 0.516	15.1 ± 0.09 ^b^	15.1 ± 0.09 ^b^	0.962
	313	Y = 0.1422Q + 0.542	14.2 ± 0.51 ^c^	14.2 ± 0.51 ^c^	0.957
Genistein	293	Y = 0.0747Q + 0.866	7.47 ± 0.11 ^d^	7.47 ± 0.11 ^d^	0.976
	303	Y = 0.0675Q + 0.881	6.75 ± 0.09 ^e^	6.75 ± 0.09 ^e^	0.981
	313	Y = 0.0661Q + 0.834	6.61 ± 0.10 ^e^	6.61 ± 0.10 ^e^	0.976

Different lowercase letters (**a–e**) after the data as superscripts in the same column indicate that the means of ANOVA with Duncan’s multiple comparison test are significantly different (*p* < 0.05).

**Table 2:** The apparent binding parameters (Ka), binding site number (n), and three apparent thermodynamic parameters for the non-covalent caseinate-polyphenol interactions at three temperatures.

Polyphenols	T (K)	n	Ka (10^5^ L/mol)	ΔH (kJ/mol)	ΔG (kJ/mol)	ΔS [J/(mol K)]
Galangin	293	1.17	8.83	11.0	−33.3	151.5
	303	1.19	10.12		−34.9	
	313	1.20	11.80		−36.4	
Genistein	293	1.04	1.02	33.4	−28.1	209.7
	303	1.08	1.54		−30.2	
	313	1.14	2.44		−32.3	

**Table 3:** Effects of sodium dodecyl sulfate (SDS) and urea on apparent binding constants (Ka) of the caseinate-polyphenol at 293 K.

Protein–Polyphenol Complex	SDS/Urea Addition	Ka (L/mol)
Caseinate-galangin	SDS	7.40 × 10^5^
	Urea	8.32 × 10^5^
Caseinate-genistein	SDS	8.60 × 10^4^
	Urea	8.83 × 10^4^

**Table 4:** The energy transfer parameters for the interaction of caseinate with galangin and genistein.

Protein–Polyphenol Complex	J (cm^3^ L/mol)	R_0_ (nm)	E	r (nm)
Caseinate-galangin	1.94 × 10^−16^	1.75	0.667	1.56
Caseinate-genistein	1.21 × 10^−16^	1.40	0.476	1.42

**Table 5:** 3D fluorescence spectral features of caseinate and caseinate-galangin/genistein systems.

Peak Parameters		Caseinate	Caseinate-Galangin	Caseinate-Genistein
Peak I	Peak position λex/λem (nm/nm)	240/345	240/350	240/350
	Fluorescence intensity	634.4	241.9	255.1
Peak II	Peak position λex/λem (nm/nm)	285/345	285/350	280/350
	Fluorescence intensity	4983	1508	2342

**Table 6:** The amino acid residues, H-bond number, and interaction energy (ΔG values) involved in the non-covalent interaction between galangin/genistein and β-casein/α_S1_-casein.

Polyphenol and Protein	Involved Residues	H-Bond Number	ΔG (kJ/mol)
Galangin and β-casein	Phe-48, Glu-51, Glu-52, Gln-55, Thr-56, Glu-57, Asp-58, Glu-59, Leu-60, Gln-61, Asp-62 *	1	−34.38
Genistein and β-casein	Ile-81, His-82, Asn-83, Ser-84, Leu-85, Arg-198, Ile-202, Pro-201, Gly-218, Pro-219 *, Phe-220, Pro-221, Ile-222, Ile-223	1	−33.41
Galangin and α_S1_-casein	Leu-3, Ile-5, Thr-7, Leu-113, Leu-116, Lys-117, Lys-120, Val-121, Thr-210, Ile-211, Ser-212, Leu-213, Trp-214	0	−34.50
Genistein and α_S1_-casein	Met-1, Leu-3, Leu-4, Ile-5 *, Thr-7, Ile-86, Leu-113, Lys-117, Lys-120, Val-121, Leu-124, Thr-210, Ile-211, Ser-212, Leu-213 *, Trp-214	2	−30.31

* Hydrogen bonding with this residue.

**Table 7:** Scavenging activities of caseinate and caseinate-polyphenol complexes to three radicals.

Protein and Its Concentration (μmol/L)	Polyphenol and Its Concentration (μmol/L)	Scavenging Activity (%)		
		DPPH Radicals	ABTS Radicals	OH Radicals
Caseinate, 15	None	26.6 ± 0.5 ^f^	19.7 ± 1.5 ^e^	41.1 ± 1.8 ^d^
Caseinate, 15	Galangin, 15	31.5 ± 0.3 ^c^	23.4 ± 0.4 ^d^	47.2 ± 0.2 ^c^
Caseinate, 15	Galangin, 25	38.1 ± 0.5 ^b^	27.2 ± 0.4 ^c^	49.2 ± 0.2 ^b^
Caseinate, 15	Galangin, 35	45.8 ± 0.1 ^a^	31.1 ± 0.3 ^a^	50.7 ± 0.4 ^a^
Caseinate, 15	Genistein, 15	27.4 ± 0.5 ^ef^	20.3 ± 0.3 ^e^	46.8 ± 0.3 ^c^
Caseinate, 15	Genistein, 25	27.9 ± 0.5 ^de^	26.8 ± 0.3 ^c^	47.9 ± 0.3 ^c^
Caseinate, 15	Genistein, 35	28.5 ± 0.5 ^d^	29.5 ± 0.2 ^b^	50.2 ± 0.4 ^ab^

Different lowercase letters (**a–f**) after the data as superscripts in the same column indicate that the means of ANOVA with Duncan’s multiple comparison test are significantly different (*p* < 0.05).

## References

[1] Corredig M., Nair P.K., Li Y., Eshpari H., Zhao Z.T. (2019). Invited review: Understanding the behavior of caseins in milk concentrates. J. Dairy Sci..

[2] Allahdad Z., Varidi M., Zadmard R., Saboury A.A. (2018). Spectroscopic and docking studies on the interaction between caseins and β-carotene. Food Chem..

[3] Farrell H.M., Jimenez-Flores R., Bleck G.T., Brown E.M., Butler J.E., Creamer L.K., Hicks C.L., Hollar C.M., Ng-Kwai-Hang K.F., Swaisgood H.E. (2004). Nomenclature of proteins of cow’s milk-sixth revisions. J. Dairy Sci..

[4] Bourassa P.C., N’soukpoé-Kossi N., Tajmir-Riahi H.A. (2013). Binding of vitamin A with milk α- and β-caseins. Food Chem..

[5] Holt C., Crver J.A., Ecroyd H., Thorn D.C. (2013). Invited review: Caseins and casein micelle: Their biological functions, structures and behavior in foods. J. Dairy Sci..

[6] Dalgleish D.G. (2011). On the structure models of bovine casein micelles-review and possible improvements. Soft Matter.

[7] Rehan F., Ahemad N., Gupta M. (2019). Casein nanomicelle as an emerging biomaterial—A comprehensive review. Colloids Surf. B.

[8] Yuksel Z., Avci E., Erdem Y.K. (2010). Characterization of binding interactions between green tea flavonoids and milk proteins. Food Chem..

[9] Khan H., Sureda A., Belwal T., Cetinkaya S., Süntar I., Tejada S., Devkota H.P., Ullah H., Aschner M. (2019). Polyphenols in the treatment of autoimmune diseases. Autoimmun. Rev..

[10] Costa C., Tsatsakis A., Mamoulakis C., Teodoro M., Briguglio G., Caruso E., Tsoukalas D., Margina D., Dardiotis E., Kouretas D. (2017). Current evidence on the effect of dietary polyphenols intake on chronic disease. Food Chem. Toxicol..

[11] Haratifar S., Meckling K.A., Corredig M. (2014). Antiproliferative activity of tea catechins associated with casein micelles, using HT29 colon cancer cells. J. Dairy Sci..

[12] Hasni I., Bourassa P., Hamdani S., Samson G., Carpentier R., Tajmir-Riahi H.A. (2011). Interaction of milk alpha- and beta–caseins with tea polyphenols. Food Chem..

[13] Sahu A., Kasoju N., Bora U. (2008). Fluorescence study of the curcumin-casein micelle complexation and its application as a drug nanocarrier to cancer cells. Biomacromolecules.

[14] Wang J., Zhao X.H. (2016). Degradation kinetics of fisetin and quercetin in solutions affected by medium pH, temperature and coexisted proteins. J. Serb. Chem. Soc..

[15] Mak K.K., Tan J.J., Marappan P., Balijepalli M.K., Choudhury H., Ramamurthy S., Pichika M.R. (2018). Galangin’s potential as a functional ingredient. J. Funct. Foods.

[16] Mukund V., Mukund D., Sharma V., Mannarapu M., Alam A. (2017). Genistein: Its role in metabolic diseases and cancer. Crit. Rev. Oncol. Hemat..

[17] Stojadinovic M., Radosavljevic J., Ognjenovic J., Vesic J., Prodic I., Stanic-Vucinic D., Velickovic T.C. (2013). Binding affinity between dietary polyphenols and β-lactoglobulin negatively correlates with the protein susceptibility to digestion and total antioxidant activity of complexes formed. Food Chem..

[18] Pu H.L., Jiang H., Chen R.R., Wang H.C. (2013). Studies on the interaction between vincamine and human serum albumin: A spectroscopic approach. Luminescence.

[19] Acharya D.P., Sanguansri L., Augustin M.A. (2013). Binding of resveratrol with sodium caseinate in aqueous solutions. Food Chem..

[20] Moeiniafshari A.A., Zarrabi A., Bordbar A.K. (2015). Exploring the interaction of naringenin with bovine beta-casein nanoparticles using spectroscopy. Food Hydrocolloid..

[21] Shu Y., Xue W.W., Xu X.Y., Jia Z.M., Yao X.J., Liu S.W., Liu L.H. (2015). Interaction of erucic acid with bovine serum albumin using a multi-spectroscopic method and molecular docking technique. Food Chem..

[22] Mehranfar F., Bordbar A.K., Parastar H. (2013). A combined spectroscopic, molecular docking and molecular dynamic simulation study on the interaction of quercetin with β-casein nanoparticles. J. Photoch. Photobio. B.

[23] Yazdi S.R., Corredig M. (2012). Heating of milk alters the binding of curcumin to casein micelles. A fluorescence spectroscopy study. Food Chem..

[24] Gao X.Y., He Y.L., Kong Y.C., Mei X.Y., Huo Y.P., He Y., Liu J.L. (2019). Elucidating the interaction mechanism of eriocitrin with β-casein by multi-spectroscopic and molecular simulation methods. Food Hydrocolloid..

[25] Morris G.M., Huey R., Lindstorm W., Sanner M.F., Belew R.K., Goodsell D.S., Olson A.J. (2009). Autodock 4 and Autodock Tools 4: Automated docking with selective receptor flexibility. J. Comput. Chem..

[26] Re R., Pellegrini N., Proteggente A., Pannala A., Yang M., Rice-Evans C. (1999). Antioxidant activity applying an improved ABTS radical cation decolorization assay. Free Radical Bio. Med..

[27] Blois M.S. (1958). Antioxidant determinations by the use of a stable free radical. Nature.

[28] Smirnoff N., Cumbes Q.J. (1989). Hydroxyl radical scavenging activity of compatible solutes. Phytochemistry.

[29] Bourassa P., Bariyanga J., Tajmir-Riahi H.A. (2013). Binding sites of resveratrol, genistein, and curcumin with milk α- and β-caseins. J. Phys. Chem. B.

[30] Li T., Hu P., Dai T.T., Li P.Y., Ye X.Q., Chen J., Liu C.M. (2018). Comparing the binding interaction between β-lactoglobulin and flavonoids with different structure by multi-spectroscopy analysis and molecular docking. Spectrochim. Acta A..

[31] Li X., Chen D., Wang G., Lu Y. (2013). Study of interaction between human serum albumin and three antioxidants: Ascorbic acid, alpha-tocopherol, and proanthocyanidins. Eur. J. Med. Chem..

[32] Sastry M.C.S., Rao M.S.N. (1990). Binding of chlorogenic acid by the isolated polyphenol-free 11 S protein of sunflower (*Helianthus annuus*) seed. J. Agric. Food Chem..

[33] Xu J.H., Hao M.H., Sun Q.F., Tang L. (2019). Comparative studies of interaction of β-lactoglobulin with three polyphenols. Int. J. Biol. Macromol..

[34] Lei S.C., Xu D.L., Saeeduddin M., Riaz A. (2017). Characterization of molecular structures of theaflavins and the interactions with bovine serum albumin. J. Food Sci. Technol..

[35] Shen F., Niu F.G., Li J.H., Su Y.J., Liu Y.T., Yang Y.J. (2014). Interactions between tea polyphenol and two kinds of typical egg white proteins-ovalbumin and lysozyme: Effect on the gastrointestinal digestion of both proteins *in vitro*. Food Res. Int..

[36] Min K., Ebeler S.E. (2008). Flavonoid effects on DNA oxidation at low concentrations relevant to physiological levels. Food Chem. Toxicol..

[37] Hatch F.T., Lightstone F.C., Covin M.E. (2000). Quantitative structure-activity relationship of flavonoids for inhibition of heterocyclic amine mutagenicity. Environ. Mol. Mutagen..

[38] Strat K.M., Rowley T.J., Smithson A.T., Tessem J.S., Hulver M.W., Liu D., Davya B.M., Davy K.P., Neilson A.P. (2016). Mechanisms by which cocoa flavanols improve metabolic syndrome and related disorders. J. Nutr. Biochem..

[39] Arts M.J.T.J., Haenen G.R., Wilms L.C., Beetstra S.A.J.N., Heijnen C.G.M., Voss H.P., Bast A. (2002). Interactions between flavonoids and proteins: Effect on the total antioxidant capacity. J. Agric. Food Chem..

[40] Niseteo T., Komes D., Belščak-Cvitanović A., Horžić D., Budeč M. (2012). Bioactive composition and antioxidant potential of different commonly consumed coffee brews affected by their preparation technique and milk addition. Food Chem..

[41] Almajano M.P., Delgado M.E., Gordon M.H. (2007). Changes in the antioxidant properties of protein solutions in the presence of epigallocatechin gallate. Food Chem..

[42] Heim K.E., Tagliaferro A.R., Bobilya D.J. (2002). Flavonoid antioxidants: Chemistry, metabolism and structure-activity relationships. J. Nutr. Biochem..

